# First reported cases of anti-NMDA receptor encephalitis in Vietnamese adolescents and adults

**DOI:** 10.1016/j.jns.2017.01.004

**Published:** 2017-02-15

**Authors:** Mai Nguyen Thi Hoang, Phu Nguyen Hoan, Tan Le Van, Angela McBride, Nghia Ho Dang Trung, Thanh Tran Tan, Hong Nguyen Thi Thu, Dorothee Heemskerk, Jeremy Day, Angela Vincent, Chau Nguyen Van Vinh, Guy Thwaites

**Affiliations:** a Oxford University Clinical Research Unit, Ho Chi Minh City, Viet Nam; b Hospital for Tropical Diseases, Ho Chi Minh City, Viet Nam; c Pham Ngoc Thach Medical University, Ho Chi Minh City, Viet Nam; d Centre for Tropical Medicine and Global Health, Nuffield Department of Medicine, University of Oxford, UK; e Department of Clinical Neurology, John Radcliffe Hospital, University of Oxford, Oxford OX3 9DU, UK

**Keywords:** Anti-NMDA, Receptor, Encephalitis, Vietnam, Adult

## Abstract

**Introduction:**

Anti-NMDA receptor encephalitis is increasingly recognised as an important differential diagnosis in patients with encephalitis of unknown aetiology. We report the first case series of patients diagnosed in Vietnam.

**Methods:**

Samples of CSF from patients with presumed encephalitis but negative microbiological investigations, who exhibited dyskinesia, autonomic instability or psychosis were tested for antibodies against the NR1 subunit of the glutamate (type-NMDA) receptor using an indirect immunofluorescence assay.

**Results:**

Of 99 patients admitted with all-cause encephalitis over an 18 month period, 9.1% (n = 9 patients, 5 female, median age 28 years) had confirmed NMDAR encephalitis. All patients were admitted from one mental health hospital, and the incidence may therefore be an underestimate. Common features included reduction in speech (n = 9), catatonia (n = 9), convulsions (n = 7), dyskinesia (n = 9), rigidity (n = 9) and autonomic dysfunction (n = 7). Aside from a modest lymphocytic pleocytosis, routine CSF analysis was usually normal. No female patient had ovarian teratoma detected by abdominal ultrasound. Most patients were treated with high dose corticosteroids, and one patient received intravenous immunoglobulin. The median duration of hospitalization was 75 days and no patient died during admission.

**Conclusions:**

Anti-NMDA receptor encephalitis is an important differential diagnosis to consider for patients presenting with acute onset psychiatric symptoms, who develop ensuing seizures, movement or autonomic disorder in Vietnam. A stronger evidence base for management and access to second line immunotherapy agents may help to reduce morbidity from this disease.

## Highlights

• Anti-NMDA encephalitis is an important differential for unexplained encephalitis.
• For the first time we have confirmed the diagnosis in adolescents and adults in Vietnam.
• Psychiatric symptoms are followed by movement/autonomic disorder and convulsions.
• Treatment is usually with high dose corticosteroids.
• A robust evidence base is required to support clinical management algorithms.

## 1 Introduction

Encephalitis is a condition defined by inflammation of the brain parenchyma with associated neurological dysfunction. Globally, its incidence has increased by 7.7% between 2005 and 2015 [1]. While encephalitis is most often suspected to be viral in aetiology, extensive investigation frequently fails to identify an infectious pathogen. Likewise in a recent cohort of 291 adults with presumed viral encephalitis admitted to our hospital, only 32% of patients had a microbiologically confirmed infection [2]. Patients with encephalitis of unknown aetiology often have prolonged hospital stays, and are discharged with lasting neurological impairment [2], [3].

Over the past 10 years, autoimmune encephalitides, especially anti-*N*-methyl d aspartate (NMDA) receptor encephalitis, have been increasingly recognised as important differential diagnoses for viral encephalitis, particularly in young adults and children [4], [5], [6]. Notably, the California Encephalitis project found that the frequency of anti-NMDA receptor encephalitis surpassed that of individual viral infections to cause 41% of known-cause encephalitis in patients aged < 30 years in the USA [7]. Since its first discovery in 2007 as a phenomenon associated with underlying ovarian teratoma, the epidemiology of anti-NMDA receptor encephalitis has shifted substantially; it has more often been reported in female patients without tumor, males and children in recent years [8]. There are few reports from resource-limited settings [9].

Distinguishing between infectious and autoimmune causes of encephalitis is essential to help direct therapy, with antimicrobial agents for the former and immunosuppression for the latter. Until recently, laboratory methods to confirm the diagnosis of anti-NMDA encephalitis were not available in Vietnam, and no cases have yet been reported from the country.

At an infectious diseases referral hospital in southern Vietnam, we investigated whether anti-NMDA receptor encephalitis was prevalent in patients with suggestive clinical features, in whom conventional microbiological testing had not identified an infectious cause. Herein we report the demographic characteristics, clinical features, management and outcomes of the first case series of patients with anti-NMDA receptor encephalitis in Vietnam.

## 2 Methods

### 2.1 Setting

The study was conducted in an adult infectious diseases ward of the Hospital for Tropical Diseases in Ho Chi Minh City, Vietnam. The ward specializes in the treatment of patients with severe central nervous system infection. The hospital is a primary, secondary and tertiary referral centre for the whole of southern Vietnam, and serves a population of over 42 million people.

### 2.2 Inclusion criteria

Primary screening criteria included adult patients (aged ≥ 15 years) admitted to the ward with presumed encephalitis, who exhibited at least one of abnormal movements (orofacial, limb or trunk dyskinesia), seizures, autonomic dysfunction and/or personality change or psychosis, and whose CSF tested negative on all microbiological investigations.

Between January 2015 and February 2016, patients meeting the screening criteria were retrospectively selected from a descriptive study aimed at improving the diagnosis of CNS infections in Vietnam. Between March and September 2016 when the diagnostic test for anti-NMDA receptor encephalitis was available as part of routine care in our hospital, any patient meeting the criteria was included.

### 2.3 Clinical information and CSF collection

Information on demographics, clinical features and management was collected, alongside an acute CSF specimen for each patient, which was either stored at − 80 °C for subsequent analysis (January 2015–February 2016) or processed immediately (March–September 2016). Routine analysis for all CSF samples included cell count, protein, lactate and glucose analysis, gram stain and bacterial culture, india ink, Ziehl-Neelson staining and real time PCR for Herpes Simplex virus 1 and 2. Additional analyses included mycobacterial culture, fungal culture, IMMY lateral Flow Assay and Japanese encephalitis virus specific IgM when clinically indicated.

### 2.4 Diagnosis of anti-NMDA receptor encephalitis

For patients selected from the descriptive study mentioned above, detection of antibodies (IgA, IgG or IgM) against the NR1 subunit of the glutamate (type-NMDA) receptor was done using an in-house [10] and/or a commercial indirect immunofluorescence cell-based assay (Cat. No. FB 112d-1005-51, EUROIMMUN, Luebeck, Germany). For the period from March to September 2016, the EUROIMMUN assay was used as part of routine diagnosis. All the assays were carried out as previously described [10] or according to the manufacturer's instructions (EUROIMMUN), and the results were read by fluorescence microscopy (Nikon). Auto-antibody testing was conducted on CSF samples only.

### 2.5 Outcome assessment and follow up

Outcomes were assessed on the basis of survival to discharge, and residual symptoms and degree of independent functioning at 8 month follow up, as reported by the patient and family members by telephone consultation.

### 2.6 Ethics

The study was approved by the Scientific and Ethical Committee of the Hospital for Tropical Diseases, Vietnam, and the Oxford University Tropical Research Ethics Committee, UK. Informed consent was obtained in writing from patients or from family members if the patient was unconscious.

## 3 Results

Of 99 patients admitted with all-cause encephalitis over the 18 month study period, 24 patients fulfilled the selection criteria. Nine patients (9.1% of patients with all-cause encephalitis) tested positive for anti-NMDA receptor encephalitis by the indirect immunofluorescence cell-based assays and were included in this case series. The demographics, clinical features, case management and outcomes are summarized in Table 1. Fifteen patients who tested negative for anti-NMDA encephalitis did not have an alternative diagnosis confirmed.

### 3.1 Patient characteristics and clinical presentation

The median age at presentation was 28 years (range 15–43 years). Five patients were female (55.6%). The median duration of hospitalization was 75 days (range 35–98 days). All the patients had been unwell for ≥ 2 weeks prior to their admission to the infectious diseases ward (median 14 days, range 14–52 days). They had all initially been admitted to a local Mental Health hospital due to the prominent psychiatric features which characterized the first few weeks of illness; these included insomnia, hallucinations, inappropriate speech, withdrawal, irritability or aggressive attacks. The development of fever, convulsions or abnormal movements prompted transfer for further medical evaluation. Subsequent clinical signs included a characteristic orofacial dyskinesia with chewing and tongue biting, which frequently resulted in traumatic injuries to the lips and tongue (Video 1, still image for Video 1). Widespread muscle rigidity was frequently accompanied by autonomic dysfunction, and eight patients required intubation due to central hypoventilation.

### 3.2 Investigations

**Fig. 1:**
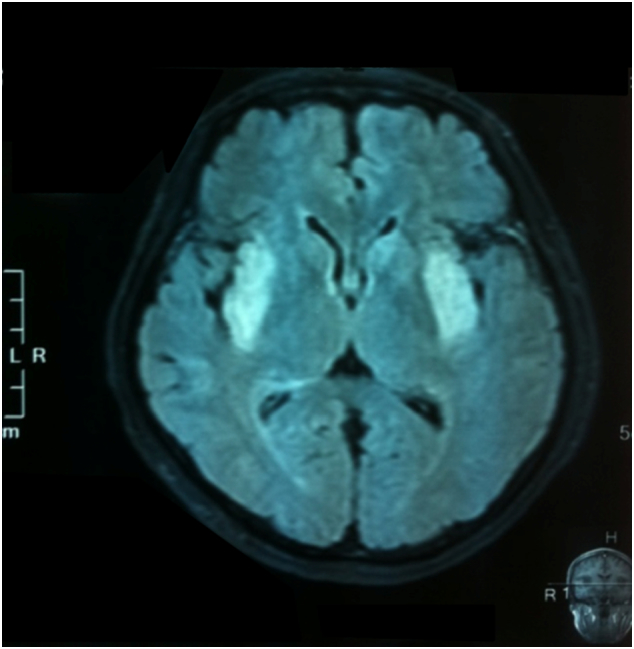
FLAIR MRI demonstrating bilateral contrast hyperintensity in the medial temporal lobes in a 44 year old male with anti-NMDA receptor encephalitis.

Diagnostic lumbar puncture was performed on all patients at the time of admission. Most patients had a modest CSF lymphocytic pleocytosis but normal protein, lactate and CSF/blood glucose ratio. All patients had intracranial imaging performed; 3 underwent CT scanning, and 6 MRI. One patient had increased signal localized to the medial aspect of both temporal lobes on T2-weighted FLAIR MRI (Fig. 1), while the imaging from the remaining eight cases was reported as normal. Trans-abdominal ultrasound was performed in all 5 female patients, but ovarian teratoma was not detected in any case.

### 3.3 Treatment and outcome

Seven patients were prescribed 1 g methylprednisolone followed by 60 mg prednisolone with weekly tapering according to clinical response. One patient was managed with an additional 5 day course of intravenous immunoglobulin (IVIG) following a failure to improve with 6 weeks of high dose corticosteroid therapy. Due to the lack of diagnostic facility at the time of enrolment, one patient did not receive any corticosteroid during their admission, and another received 16 mg dexamethasone for 7 days. Convulsions and rigidity were managed with phenobarbitol and diazepam as first-line agents.

Eight patients had survived to discharge at the time of writing; a final patient remained in hospital. Three required further admission to a rehabilitation facility prior to discharge home. Seven patients have been followed up at 8 months: relatives reported that all patients exhibited behavioural disturbance and/or cognitive deficit for many weeks following their discharge from hospital. However at 8 months, four patients had returned to work/school, and the remainder were able to perform activities of daily living, although two had residual cognitive deficits and required prompting to initiate these tasks.

## 4 Discussion

Herein we have described the first case-series of anti-NMDA receptor encephalitis diagnosed in Vietnam.

Consistent with other published case series, we found that our patients were young adults, but we did have a higher proportion of males than in early reports [6]. This may reflect increased recognition that the disease need not be associated with underlying teratoma, and is consistent with the recent shift in disease epidemiology. As has been previously described, most patients had a modest CSF pleocytosis but normal protein and glucose levels [6]. All but one of our patients had intracranial imaging within normal limits. The finding of contrast enhancement in the temporal region on T2 weighted MRI has been reported previously; it was noted in 16 of 55 patients with MRI abnormalities in a cohort of 100 patients with anti-NMDA receptor encephalitis [6].

There was no evidence for underlying teratoma in our five female patients, which may reflect the small sample size or the diagnostic methods used; we performed trans-abdominal ultrasound to screen patients, rather than more sensitive imaging such as trans-vaginal ultrasound, CT or MRI [11]. Additionally, some patients have had tumors detected only on histological examination of prophylactically excised ovaries [12], [13], or developed detectable teratoma many months into convalescence from NMDA receptor encephalitis [5], [6]. Of note, the detection and removal of teratoma is associated with more favourable outcomes in anti-NMDA receptor encephalitis [6], [10] [14] which highlights the need to use both more sensitive methods to screen patients, and to ensure follow up imaging for those who do not have detectable tumor during the acute phase.

All patients who had their diagnosis confirmed during admission received high dose methylprednisolone, followed by tapered oral prednisolone. Two patients who were not managed as such were among those diagnosed retrospectively by analysis of stored CSF. The final patient in our case series has also received a 5 day course of IVIG. However IVIG is prohibitively expensive for the majority of patients in Vietnam. Plasma exchange and second line immunotherapies such as rituximab and cyclophosphamide are not available in our setting, and although observational data report more favourable outcomes in patients who have received them, there are no randomized clinical trials to support their routine use [8]. Although 8 of 9 patients have survived to discharge and there have been no reported relapses, the long duration of hospitalization, compounded further by the loss of earnings due to the extended recovery phase contributes to substantial morbidity and places a potentially catastrophic financial burden on affected patients and their families. Robust, evidence based management algorithms are urgently required for patients with anti-NMDA receptor encephalitis. These in turn may help clinicians, especially in resource limited settings, advocate for access to currently unavailable therapies for this treatable condition.

It is striking that all of our patients were admitted from one Mental Health hospital. It suggests the true burden of anti-NMDA receptor encephalitis in Vietnam may be significantly greater than is represented by our case series. It is vital that clinicians evaluating patients who present with psychiatric symptoms in Vietnam are aware that anti-NMDA receptor encephalitis is a differential diagnosis, and assess recently admitted patients regularly for the development of movement disorder, convulsions or autonomic dysfunction such that diagnosis and immunotherapy can be initiated as soon as practicable. Of note, it has been shown that both short and long term outcomes appear to be adversely affected by delay between symptom onset and starting therapy [15], [8].

For the first 12 months of this study, consensus criteria for the diagnosis of probable anti-NMDA receptor encephalitis were not available. As such, we attempted to be inclusive in our identification of patients for anti-NMDA receptor antibody testing. Criteria were published to aid diagnosis and commencement of immunotherapy in the absence of antibody testing results in February 2016 [16], and these may be particularly helpful to clinicians working in resource-limited settings, where the diagnostic facility to confirm anti-NMDA receptor encephalitis may not yet be available.

The absence of children from the case series, and the investigative focus on anti-NMDA receptor encephalitis alone are other limitations of this study. Going forward, we need to assess the burden of childhood encephalitis caused by anti-NMDA receptor encephalitis in Vietnam as a priority, and complement this study with a panel of assays searching for other antibodies against neuronal cell surface antigens including AMPAR, GABAbR, GABAaR, LGT1 and Caspr2, to help improve understanding of the epidemiology and management of non-infectious encephalitis in the region.

## 5 Conclusion

Anti-NMDA receptor encephalitis is an important differential diagnosis to consider for patients presenting with acute onset psychiatric symptoms, and for patients admitted to hospital with encephalitis of unknown aetiology in Vietnam. The suggestive clinical history and ensuing movement/autonomic disorder should prompt auto-antibody testing, such that immunotherapy can be initiated at the earliest opportunity. Sensitive imaging modalities such as MRI should be employed to search for underlying teratoma, and negative patients followed up in convalescence. A stronger evidence base for management is urgently needed to help clinicians in resource limited settings advocate for improved access to second line immunotherapy agents.

The following is the supplementary data related to this article.

Video 1 Orolingual dyskinesia with tongue involvement and bite injuries in an adult male patient with anti-NMDA receptor encephalitis. Video 1

## Funding

This work was supported by the Wellcome Trust, UK [101104/Z/13/Z and 106680/B/14/Z]. The funding body did not have any influence on the study design, study conduct, preparation of the manuscript or decision to publish.

## Conflicts of interest

None.

## Figures and Tables

**Table 1:** Clinical features displayed by patients with confirmed anti-NMDA receptor encephalitis at any stage during their hospital admission.

Characteristic	
Female	5 (55.6)
Age	28 (15–43)^
Duration of illness prior to admission to HTD (days)	14 (14–52)^
Prior admission to Mental Health hospital	9 (100)
Symptoms	
Insomnia	2 (22.2)
Delusions	2 (22.2)
Reduction in speech/mutism	9 (100)
Irritability	4 (44.4)
Hyperactivity	4 (44.4)
Catatonia	9 (100)
Clinical signs	
Fever > 38 °C	2 (22.2)
Convulsions	7 (77.8)
Movement disorder	
Chewing	8 (88.9)
Tongue biting	5 (55.6)
Dystonia	5 (55.6)
Rigidity	9 (100)
Autonomic dysfunction	
Tachycardia	7 (77.8)
Tachypnoea	7 (77.8)
Sweating	7 (77.8)
Required invasive ventilation	8 (88.9)
CSF analysis	
CSF white cell count (cells/mm^3^)	16 (5–116)^
CSF protein (g/dL)	0.24 (0.2–0.6)^
CSF glucose (mmol/L)	4.1 (3.5–5.3)^
CSF glucose/blood glucose ratio > 0.5	9 (100)
CSF lactate (mmol/L)	1.9 (1.7–3.3)^
Imaging	
CT	3
MRI	6
Reported as abnormal (see Fig. 1)	1
Investigation for teratoma	
Trans-abdominal ultrasound (female)	5
Management	
Methylprednisolone/prednisolone	7
Intravenous immunoglobulin	1
Dexamethasone	1
No corticosteroid therapy	1
Outcome at discharge (n = 8)	
Survival to discharge	8
Duration of hospital stay (days)	75 (35–98)^
Outcome at 8 months (n = 7)	
Returned to work/school	4
Independent with activities of daily living	7
Residual cognitive deficit	2

N (%), Median (range)^. Unless otherwise stated, n = 9.
