# Supplementary figures and images for: First reported cases of anti-NMDA receptor encephalitis in Vietnamese adolescents and adults
Source: J Neurol Sci. 2017 Feb 15;373:250–3. doi: 10.1016/j.jns.2017.01.004 (PMC5293131; doi:10.1016/j.jns.2017.01.004)

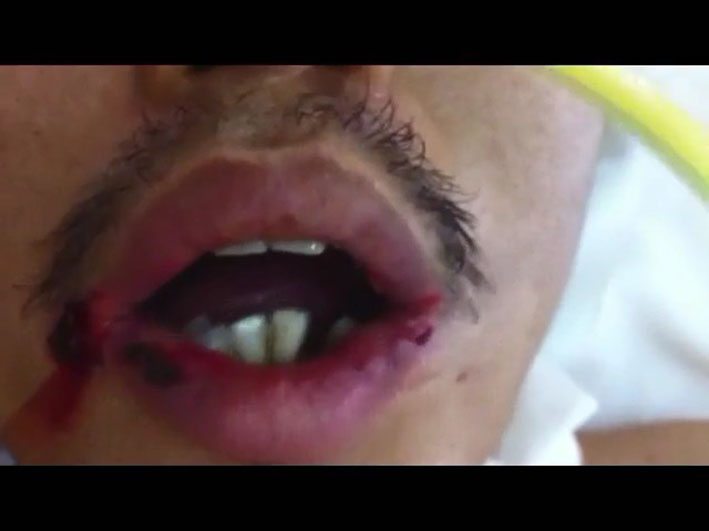

Supplement: Video 1 — Orolingual dyskinesia with tongue involvement and bite injuries in an adult male patient with anti-NMDA receptor encephalitis. [file mmc1.jpg]
